# Evaluating the use of programmed reinforcement in a correction procedure with children diagnosed with autism

**DOI:** 10.1186/s41155-019-0134-3

**Published:** 2019-11-15

**Authors:** Ana Carolina Cabral Carneiro, Eileen Pfeiffer Flores, Romariz da Silva Barros, Carlos Barbosa Alves de Souza

**Affiliations:** 1 Universidade Federal do Pará, Belém, PA Brazil; 2 Universidade de Brasília, Brasília, Brazil; 3 Instituto Nacional de Ciência e Tecnologia sobre Comportamento, Cognição e Ensino, Sao Carlos, Brazil

**Keywords:** Correction procedure, Reinforcement, Verbal behavior, Autism

## Abstract

**Background:**

Procedures that reduce errors while learning a repertoire play an important role in Applied Behavior Analysis for people with autism due to the detrimental effects that excessive exposure to error may have on learning. Previous studies have investigated the effects of correction procedures that require active student response after a trial with error. Some intervention manuals recommend against reinforcing responses after correction to prevent the establishment of prompt dependence. This study directly investigated the effect of reinforcement after an active-response correction procedure during tact training in four children with autism. An echoic-to-tact training procedure was used to train tacts. A “no reinforcement after correction” (NRC) condition was compared to a “reinforcement after correction” (RC) condition, using an adapted alternated treatments design.

**Results:**

All participants needed less correction trials in RC than in NRC, and considering all 26 sessions in which both training procedures were implemented, participants’ performance was higher with RC than without in 17 sessions and was the same in 3 sessions.

**Conclusions:**

We discuss the effectiveness of reinforcing correct responding after an active-response correction procedure, the absence of prompt dependence, and the implications of better correction procedures for applied settings.

## Background

An important aspect of effective autism intervention is the design of procedures that minimize errors, since these may lead to performance deterioration (Greer and Ross, 2008; Lovaas, 2003; Mueller, Palkovic, and Maynard, 2007). Although errors are often inevitable, some features of the teaching procedures may be relevant to make them less frequent.

Correction procedures are recommended in autism intervention handbooks (e.g., Greer and Ross, 2008; Maurice, Green, and Foxx, 2001; Sundberg and Partington, 1998). Several correction procedures have been proposed and investigated. In some procedures, incorrect responses are followed by error statements (saying “no” to the incorrect response) or modeling the correct response, without requiring an active learner response (Barbetta, Heron, and Heward, 1993; McGhan and Lerman, 2013; Smith, Mruzek, Wheat, and Hughes, 2006). In other correction procedures, an active learner response is required after a corrective trial, such as in single-response repetition procedure (the correct response is modeled, and the participant is required to repeat the correct response), remove and re-present (after the incorrect response a brief time-out is implemented and the trial is re-presented with the correct response modeled) and directed rehearsal (the correct response is modeled and the trial is re-presented until a specific number of correct unprompted responses occur) (Barbetta, Heward, and Bradley, 1993; Barbetta, Heward, Bradley, and Miller, 1994; Carroll, Joachim, St. Peter, and Robinson, 2015; Carroll, Owsiany, and Cheatham, 2018; Kodak et al. 2016; Rapp et al. 2012; Rodgers and Iwata, 1991; Turan, Moroz, and Croteau, 2012; Worsdell et al. 2005).

Taken together, studies point to the effectiveness of multiple error-correction procedures and suggest that the efficiency of each procedure varies according to the characteristics of the learners and correction procedures used (Carroll et al. 2015; Carroll et al. 2018; Kodak et al. 2016; Ingvarsson and Hollobaugh, 2011; Ingvarsson and Le, 2011; Kodak, Fuchtman, and Paden, 2012; McGhan and Lerman, 2013).

Concerning the correction procedures with an active learner response requirement (usually recommended in manuals for autism intervention, e.g., Greer and Ross, 2008; Maurice et al. 2001) studies have evaluated, with mixed results, two main effects. The first one is the effect of requiring more or fewer repetitions of the corrective trial (i.e., more or fewer opportunities to actively practice the response, e.g., Cuvo, Ashley, Marso, Zhang, and Fry, 1995; Marvin et al. 2010; Worsdell et al. 2005). The second effect is that of inserting a mastered target between a prompted trial (after an error) and the opportunity for independent response, to prevent prompt dependence (Plaisance, Lerman, Laudont, and Wu, 2016; Turan, Moroz, and Croteau, 2012). Apparently, independently of specific features of the procedure, the effectiveness of correction procedures in such cases may be related to negative reinforcement (Rodgers and Iwata, 1991). Correction procedure may be aversive and correct responses may function to avoid correction (but see Plaisance et al. 2016).

However, a more basic question concerning correction procedures with an active learner response requirement has not been investigated to date: the effect of reinforcement for correct responses on corrective trials (after an error). Overall, studies have presented praise as a consequence for correct responses in prompted correction trials, keeping edible/tangible items (supposedly high-magnitude reinforcers) for independent responses (e.g., Carroll et al. 2015; Carroll et al. 2018; Kodak et al. 2016; Rapp et al. 2012; Turan et al. 2012). Handbooks focused on interventions for people with autism (e.g., Greer and Ross, 2008) often caution against using programmed reinforcement to correct active responding on a corrective trial that is presented after an error. The recommendation seeks to avoid possible prompt dependent behavior that could arise in correction procedures. It is assumed that this prompt dependent behavior can be established as a consequence of a higher reinforcement density, product of corrected and independent responses reinforcement, or of the delayed reinforcement effect of the incorrect responses that precede the strengthening of corrected responses (Catania, 1971; DeLeon, Bullock, and Catania, 2013).

Nevertheless, studies discussing possible prompt dependence (Cividini-Motta and Ahearn, 2013; Karsten and Carr, 2009; Vladescu and Kodak, 2010) have focused on the effects of cues or prompts used in errorless learning procedures without addressing the issue of reinforcement on corrective trials. Therefore, the recommendation to avoid programmed reinforcement for correct responding in corrective trials needs empirical support.

The present investigation addresses this issue directly. We conducted an experimental investigation of the effect of reinforcing active responding in corrective trials while teaching tacts^1^ to children diagnosed with autism. We used an adapted alternating treatments design (Sindelar, Rosenberg, and Wilson, 1985) to contrast correction with and without programmed reinforcement during tact acquisition.

## Method

### Participants

Four boys (aged 4 to 8 years) diagnosed with autism participated. We assessed participants’ verbal repertoire using the Verbal Behavior Milestones Assessment and Placement Program (VB-MAPP–Sundberg, 2008). All children reached level 1 abilities (0–18 months) and at least some level 2 abilities (18 to 30 months). All four children were enrolled in mainstream classrooms. Participant 1 (aged 4 years) and participant 4 (aged 6 years) were enrolled in a university-based project that provided training for caregivers of children diagnosed with autism. Participant 2 (aged 8 years) and participant 3 (aged 5 years) were enrolled in the same project and additionally received approximately 5 h per week of behavior-analytic intervention. Participants 1 and 2 had previously undergone occupational and speech therapy in other institutions.

### Ethical considerations

This study was approved by the Research Ethics Committee at the Health Sciences Institute, Federal University of Pará (Consent Number 175.303). Participants’ caregivers signed an informed consent authorizing children’s participation in the study.

### Setting and materials

Sessions were conducted at the university, in a room (36 m^2^) prepared and designated for research and intervention with children diagnosed with autism. Sessions were filmed using a Sony HDD DCR-SR87 camera and target behaviors were recorded using customized sheets.

### Discriminative stimuli

**Fig. 1:**
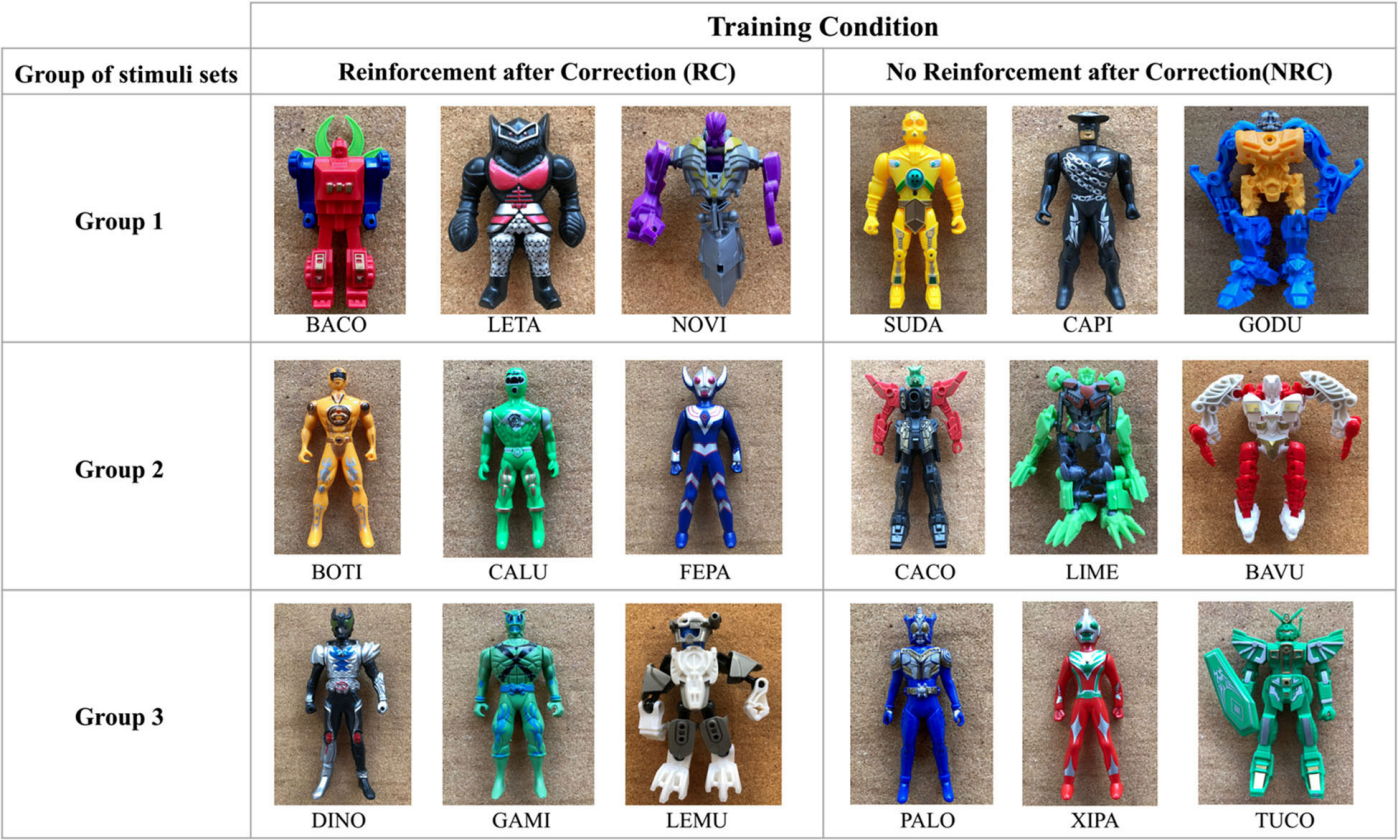
Stimulus sets by group of training in RC and NRC conditions.

Stimuli were 18 anthropomorphic tridimensional objects, divided into 3 groups with 2 sets of 3 stimuli each (see Fig. 1). Stimuli were named with two-syllable contrived words (e.g., *Baco*, *Gami*, *Lemu*) to minimize uncontrolled effects of children’s learning history regarding the stimuli.

### Consequence stimuli

We interviewed caregivers about possible reinforcers and conducted daily multiple-stimulus-without-replacement preference assessments (Carr, Nicolson, and Higbee, 2000) to rank items by preference (see DeLeon and Iwata, 1996 on correlations between preference and reinforcing function). Praise and approval were also used (e.g., “Well done!” “Cool!”).

### Experimental design

We used an adapted alternating treatments design (Sindelar et al. 1985) to evaluate the effect of reinforcement after correction on tact acquisition. Each participant was exposed to alternating treatments using two sets of three stimuli. For one set, we used an active-response correction procedure followed by reinforcement of correct responses (“reinforcement after correction” condition—NRC). For the other set, there was no reinforcement for corrected responses (“no reinforcement after correction” condition—NRC). Dependent variables were count of tacts learned, sessions to mastery criterion, and number of correction trials in each condition. Order of conditions was balanced between participants.

### Procedure

Sessions were conducted 2–3 times a week. Initially, we assessed which syllables each child could pronounce and used them to compose the two-syllable-contrived stimulus names.

### Assessment of syllable pronunciation

An assessment session consisted of 20 trials in which the experimenter asked the child to imitate vocal one-syllable stimuli (e.g., “*ba*”). Tangible and social reinforcements followed correct responses. Errors or failure to respond after 5 s led to a new trial. Sessions were continued until at least 10 syllables were found that the child could reproduce correctly.

### Tact training

Tacts were taught using an echoic-to-tact training procedure (Greer and Ross, 2008)^2^. Each session consisted of 12 trials with one of the 3-stimuli sets (4 trials per stimulus). Stimuli were presented in random order. Inter-trial interval varied depending on the reinforcer presented on the previous trial.

**Fig. 2:**
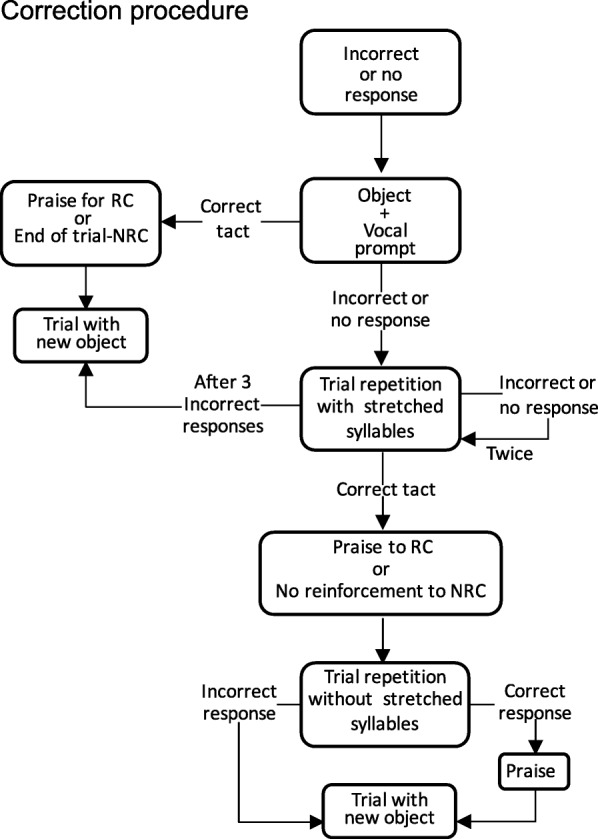
Correction procedure flowchart. NR = no reinforcement after correction condition. RC = reinforcement after correction condition

The training was implemented in two stages (training with vocal prompt; and training with delayed vocal prompt). The first training stage used simultaneous vocal prompts. Each trial began with the experimenter presenting a tridimensional stimulus and saying: “[stimulus name]. What’s this called?” or “[stimulus name]. What´s this?” (The experimenter alternated the two question formats). Correct responses led to social and tangible reinforcement (with a low-magnitude reinforcer, as previously defined by the multiple-stimulus preference assessment) and ended the trial. Incorrect responses or failure to respond after 5 s led to the correction procedure (see Fig. 2).

Immediately after an incorrect response, the experimenter showed the stimulus and repeated the prompt as described above. A correct response ended the trial in the NRC condition or led to social reinforcement and the end of the trial in the RC condition. If the participant gave an incorrect response or did not respond after 5 s, the experimenter repeated the trial, this time stretching out the syllables of the vocal prompt. If the participant responded incorrectly or failed to respond, the experimenter repeated the procedure up to two more times, stretching out the syllables of the vocal prompt. If the participant did not respond correctly after the third repetition, the trial was ended. Correct responses at this point led to social reinforcement and repetition of the trial without stretching out the syllables of the vocal prompt (in RC condition) or just to a repetition of the trial without the stretching-out procedure (in NRC condition). Correct or incorrect response ended the trial, and correct response resulted in social reinforcement in RC condition.

The learning criterion in this first stage was at least three correct responses out of four trials for each stimulus (i.e., at least 9 correct responses in 12 trials and at most 1 incorrect response for each stimulus).

Once a participant reached the learning criterion for one of the stimuli with vocal prompt, training for that stimulus proceeded to a second stage in which a 5-s delay was inserted between stimulus presentation and vocal prompt. The experimenter presented the stimulus and, in alternating trials, asked “What’s this called?” or “What’s this?” and waited for 5 s. In other trials, the experimenter simply presented the stimulus and waited for 5 s. In either case, correct responses were given before the vocal prompt lead to social and tangible reinforcement (with a higher-magnitude reinforcer, as previously defined by the multiple-stimulus preference assessment) and ended the trial. If the child emitted an incorrect response or did not respond after 5 s, the experimenter began the same correction procedure used in the previous training phase. The same learning criterion was used as in the previous stage (minimum of 9/12 correct responses with maximum one error per stimulus).

After three consecutive sessions, the experimenter began training with a new set of three stimuli, whether or not the child had reached criterion for all stimuli of the previous set. This was done to avoid excessive exposure to error.

### Inter-observer agreement and treatment integrity

A second experimenter independently observed the filmed records of 30% of experimental sessions and scored performance in order to establish inter-observer agreement ([agreements/disagreements + agreements] × 100). The second observer also judged treatment integrity by observing whether all planned procedures were correctly and thoroughly implemented ([correct procedures/planned procedures] × 100 for stimulus presentation, consequence presentation, and correction procedures). Inter-observer agreement for performance scores varied between 93.5% and 100%. Treatment integrity for stimulus presentation was 100% for all four participants. Treatment integrity for consequences and for correction procedures was 100% for participants 1, 3, and 4 and 94 % for participant 2.

### Results

During the initial tact training stage, with simultaneous vocal prompts, all four participants reached 100% correct responses in one session. Therefore, correction procedures were unnecessary at this stage.

Total percentage of correct tacts in the training stage with delayed vocal prompts was 32.81% for stimulus set used in NRC Condition, and 50.55% for stimulus set used in RC condition. Mean trials to criterion for each stimulus were 4.58 in the NRC condition and 2.5 in the RC condition for participant 1; 5.25 in the NRC and 4.5 in the RC condition for participant 2; 2.5 in NRC and 1.67 in RC for participant 3; 8.75 in NRC and 5 in RC for participant 4.

**Fig. 3:**
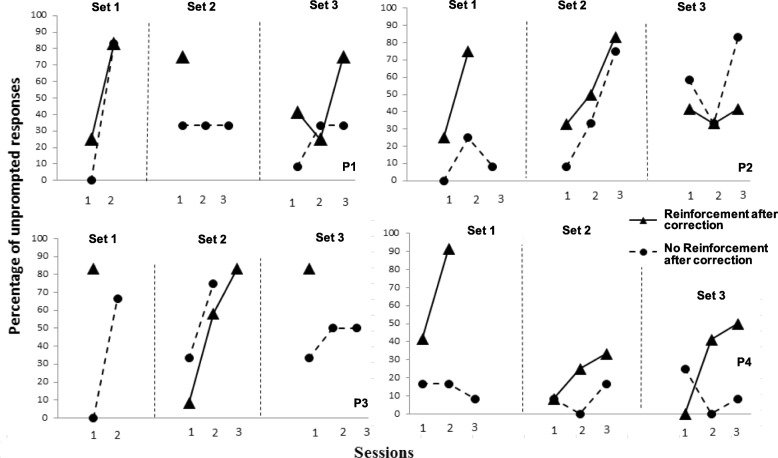
Percent unprompted correct responses per session for three trained sets of tacts (set 1, set 2, and set 3) for each participant (P1, P2, P3, and P4), during training sessions with delayed echoic prompts, with and without reinforcement for correct answers after correction procedures Endnotes ^1^Tact: A verbal operant controlled by non-verbal antecedent stimuli and maintained by generalized reinforcement (Skinner, 1992). For example, a child sees a toy bear and says “Bear.” ^2^Echoic: a verbal operant controlled by verbal stimuli and maintained by generalized reinforcement, in which there is point-to-point correspondence and formal similarity between antecedent and response (Skinner, 1992). For example, a child hears her father say “Good girl” and she repeats “Good girl.”

Figure 3 shows the percentage of unprompted correct responses per session, for all three sets of trained tacts, for each participant, with (RC condition) and without reinforcement (NRC condition) for correct responses after correction procedures. Taking into account all 26 sessions in which both training procedures were implemented, participants’ performance was higher with RC than without in 17 sessions and was the same in three sessions, i.e., performance tended to be more accurate when reinforcement was delivered contingent on correct responses after correction procedures.

Considering participants’ performance with each set of stimuli, it is possible to observe that participant 1 reached the learning criterion for set 1 during the second session, for both conditions. For set 2, he reached criterion in only one session in RC but did not reach criterion after three sessions in NRC, and for set 3 he reached criterion during the third session with RC but did not reach criterion after three sessions in NRC.

Participant 2 reached the learning criterion for set 1 at the end of the second session in the RC condition but did not reach criterion after 3 sessions in the NRC condition. For set 2, he reached criterion after 3 sessions in both conditions, and for set 3 he did not reach criterion after three sessions in RC and reached criterion during the third session in NRC.

Participant 3 needed only one session to reached learning criterion for set 1 in the RC condition, but did not reach learning criterion after two training sessions in NRC (due to experimenter error, a third session was not held in this condition). For set 2, he required three sessions to reach criterion in RC and two sessions in NRC, and for set 3 he needed only one session to achieve the learning criterion in RC but did not reach criterion after three sessions in NRC.

Participant 4 reached the learning criterion for set 1 in two sessions in RC condition but did not reach learning criterion at the end of three training sessions in NRC. For sets 2 and 3, in both conditions, participant 4 did not reach criterion, but performance was superior in the RC condition for four out of six sessions.

Table 1 presents the frequency and level of correction trials needed in RC and NRC conditions. All participants needed a higher frequency of correction trials when there was no reinforcement after correction (NRC) than when there was (RC). Three participants (P1, P2, and P4) also needed correction beyond the first delayed vocal prompt (stretched-out vocal prompts) in the NRC condition, but not in the RC condition.

**Table 1:** Level and frequency of correction needed in RC and NRC conditions for each participant

	Reinforcement after correction (RC)				No reinforcement after correction (NRC)			
	Participants							
Level of correction	P1	P2	P3	P4	P1	P2	P3	P4
Delayed vocal prompt	30	51	20	58	47	59	30	94
Stretched-out vocal prompt 1	0	3	0	2	4	4	0	4
Stretched-out vocal prompt 2	0	0	0	0	4	0	0	7

## Discussion

This study investigated the effect of reinforcement after correction with active response procedure on the learning of tacts by children with autism. A correction trial requiring an active response was presented immediately after each error (Worsdell et al. 2005; Barbetta et al. 1994). This procedure was compared with another in which reinforcement did not follow corrected responses, as recommended in some intervention handbooks (e.g., Greer and Ross, 2008; Maurice et al. 2001).

In general, data from the present study suggest that delivering reinforcement contingent on correct responses during correction trials can benefit tact acquisition in children with autism. Performance was generally superior when prompted responses were corrected, as measured by attainment of learning criterion, trials or sessions to criterion, percent correct responses, as well as frequency and level of correction necessary for learning. Of course, it is possible that difference in participants’ performance between RC and NRC conditions could diminish with additional training, considering that training with each set of stimuli ended after three sessions (even though performance was quite low in both conditions—see participant 4’s performance). However, this does not affect the result that, in a general way, participants needed fewer trials or sessions, with lower frequency and level of correction, to criterion in RC condition, which has implications when planning more effective interventions for people with autism.

The results of this study are at odds with guidelines presented in some handbooks of Applied Behavior Analysis (e.g., Greer and Ross, 2008; Maurice et al. 2001) which recommend against reinforcing responses on correction trials. Conceivably, such guidelines should be derived from research showing that prompting procedures can lead to prompt dependency, since prompting is often involved in correction procedures, but as mentioned before, studies discussing possible prompt dependence (e.g., Cividini-Motta and Ahearn, 2013; Karsten and Carr, 2009; Vladescu and Kodak, 2010) have focused on the effects of prompts in errorless learning procedures, without addressing the issue of reinforcement on corrective trials. The present study directly investigated the effect of reinforcement after an active-response correction procedure during tact training in children with autism, and the results do not offer empirical support for claims that reinforcement of correct responding on correction trials is detrimental to performance.

As previously indicated, it is assumed that prompt dependent behavior can be established as a consequence of a higher reinforcement density, as a product of reinforcement of corrected as well as independent responses or through the delayed reinforcement effect of incorrect responses that precede the strengthening of corrected responses (Catania, 1971; DeLeon, Bullock, and Catania, 2013). The results of the present study also do not support this assumption, but considering that parametric manipulations of the magnitude of the reinforcers have not been performed, new studies must be carried out to evaluate this question more systematically.

The adapted alternating treatments design used in this study (Sindelar et al. 1985) allowed us to evaluate the efficacy of two different procedures on correction trials on the acquisition of tacts. However, with this type of alternating treatment design it is not possible to rule out completely carryover effects from one treatment to the other. A replication of the present study using a sequential alternating treatment design (Wacker et al. 1990) with order presentation counterbalanced across-subjects is therefore in order.

Further development of this line of research may contribute to our understanding of the effects of reinforcement after correction procedures on teaching other verbal repertoires for children with autism, as well as with other correction procedures reported in the literature (e.g., directed practice, response repetition—Carroll et al. 2015; McGhan and Lerman, 2013; Rapp et al. 2012). Future studies may also include follow-up measures to assess the long-term effectiveness of the procedures investigated in this study. Besides that, considering that during the correction procedure in the present study it was used as reinforcer praise only (in line with previous research, e.g., Carroll et al. 2015; Carroll et al. 2018; Kodak et al. 2016; Rapp et al. 2012; Turan et al. 2012), futures studies should evaluate situations in which the use of social reinforcement alone in correction procedures is not sufficient and it is necessary to include tangible reinforcers in the teaching procedure.

## Conclusions

The results from this study suggest that delivering reinforcement contingent on correct responses during correction trials can benefit tact acquisition by children with autism. These results may be useful for planning minimal-error interventions designed for children with autism, thus avoiding the detrimental effects of exposure to repeated error (Greer and Ross, 2008; Lovaas, 2003; Mueller, Palkovic, and Maynard, 2007). The procedure may also be helpful when planning assessment and instruction strategies in other contexts, such as the classroom.

## Data Availability

The datasets used and/or analyzed during the current study are available from the corresponding author on reasonable request.

## Abbreviations

NRC: No Reinforcement after Correction Condition
RC: Reinforcement after Correction Condition
VB-MAPP: Verbal Behavior Milestones Assessment and Placement Program
